# **BELFAST **nonagenarians: nature or nurture? Immunological, cardiovascular and genetic factors

**DOI:** 10.1186/1742-4933-7-6

**Published:** 2010-05-27

**Authors:** I M Rea

**Affiliations:** 1 Department of Geriatric Medicine, School of Medicine, Dentistry and Biomedical Science, Queens University Belfast, Whitla Medical Building, 97 Lisburn Road, Belfast BT9 7BL, Northern Ireland

## Abstract

Nonagenarians are the fastest growing sector of populations across Western European and the developed world. They are some of the oldest members of our societies and survivors of their generation and may help us understand how to age not only longer, but better.

The Belfast Longevity Group enlisted the help of 500 community-living, mobile, mentally competent, 'elite' nonagenarians, as part of an ongoing study of ageing. We assessed some immunological, cardiovascular, nutritional and genetic factors and some aspects of their interaction in this group of 'oldest old'.

Here we present some of the evidence related to genetic and nutritional factors which seem to be important for good quality ageing in nonagenarians from the **B**elfast **E**lderly **L**ongitudinal **F**ree-living **A**geing **ST**udy (**BELFAST**).

## Background

Nonagenarians are the oldest members of our societies and survivors of their generation. But what are the factors which help people reach their ninetieth birthday in good condition? Are they genetics as in '*nature*' or do they depend on '*nurture*' and are related to environment, or are both factors inextricably intertwined?

The Belfast Longevity Group has argued that in order to age successfully, it seems necessary to have a combination of facilitatory genes and appropriate nutrition to drive

a) a competent immune system, and provide b) a favourable cardiovascular risk profile

Here we present some of the evidence related to genetic and nutritional factors which seem to be important for good quality ageing in nonagenarians from the **B**elfast **E**lderly **L**ongitudinal **F**ree-living **A**geing **ST**udy (**BELFAST**).

### BELFAST Study

#### Subjects and Methods

In the **BELFAST **study, 90 year old subjects, who are '*very good*' for their age, also called '*elite*' (approximately 500), were recruited through their General Practitioners, from the Greater Belfast area [1,2]. Subjects willing to enrol, were community-living, mobile, and mentally competent (> 26/30), [3] and gave written Ethical Consent, Queens University Belfast. Briefly, subjects gave blood samples for DNA and other laboratory variables, responded to nutrition, life style and medical history questionnaires and had Blood Pressure and anthropometric measurements taken [2,4].

### Immunology of **BELFAST** Nonagenarians

An effective immune system needs 3 major components which are influenced by both nutrition and by genes

• Innate Immune Response - neutrophils and natural killer cells (NK)

• Cell-mediated Immunity - through lymphocyte subsets research

• Cytokines - the immune hormonal messengers

### Innate Immune System

#### Neutrophil Function

In early work on neutrophils, we showed that chemotaxis appeared unchanged in '*elite*' nonagenarian subjects compared to younger people. In terms of neutrophil engulfment, phagocytosis was modestly reduced in older males compared to female nonagenarians and younger subjects, Table 1. Killing of Staphylocccus aureus was equal across both age groups though myeloperoxidase activity, a measure of oxidative killing capacity, appeared none significantly lower in older women [5]. However in general, there is a dearth of information about neutrophil function with respect to ageing, and what information is available has been somewhat contradictory and difficult to interpret [6-8]. Some groups have reported similar findings to those of the BELFAST study in that centenarians seem to have better neutrophil function compared to those in the 'younger' old groups [9], and normal chemotaxis has been associated with better survival [10]. However neutrophil immunology has been relatively under researched and much further work requires to be carried out in this area.

**Table 1:** Neutrophil Phagocytosis of Staphylococcus aureus

Elderly Male	61%
Young Male	64%
Elderly Female	70%
Young Female	66%
	P = 0.05

Adapted from Rea IM, 1988

#### Natural Killer Cells (NK) and NKT-related cells

Natural killer cells provide the highly important innate immune response which is first-line and widely conserved thorough out nature. Their role is to target and kill virally infected, tumorigenic or other abnormal cells. They kill by releasing cytotoxic molecules, stored in cytoplasmic secretory lysosomes. NK cell numbers have been noted to be increased in many studies related to ageing [11-15], where it is thought that they scan the immunological landscape and deal with the increased susceptibility to tumourigenesis, which comes with increasing age. Like other studies in the oldest old, BELFAST nonagenarians showed increased numbers of NK cells [16].

However, the BELFAST Longevity Group, was the first to identify increases in other NKT-related cells [16] which are now considered to act as first responders and serve as a bridge between the innate and adaptive immune system. NKT cells recognize lipid antigens rather than peptides, and respond to these when presented by a non-classical class I MHC molecule, CD1d.

NKT cells constitute a minor lymphocyte population that exhibits features of both T cells and NK cells. They seem to play a pathogenic role in disease, have immune-regulatory properties, in part based on their cytokine profile, though in cancer, there is evidence that they can both activate or suppress anti-tumour immunity [17]. Recent research on the CD56 (bright) NK cell subset confirms that these cells are numerically in the minority in peripheral blood but constitute the majority of NK cells in secondary lymphoid tissues. Although increased in ageing, the actual role of NKT cells is not yet completely understood, though like NK cells, they seems to be important in first-line immune surveillance though perhaps against a different spectrum of abnormal cells.

#### NK cells and Nutrition

**Figure 1:**
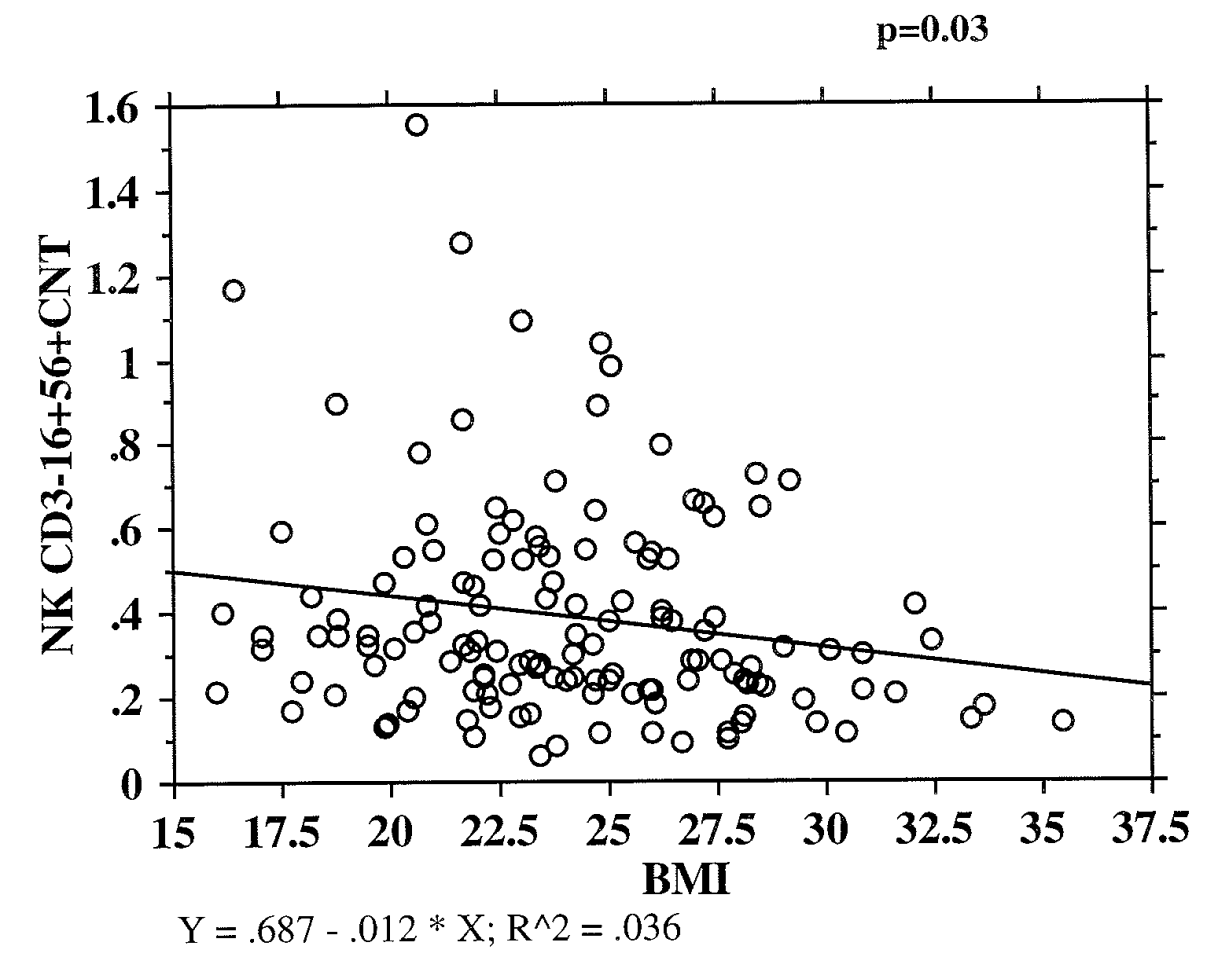
**NK cells and BMI in BELFAST study**.

NK cells and their cytolytic activity have been related to nutrition including Vitamin D and anthropometric markers [18]. In preliminary work, there appears to be a small negative relationship between NK cell number and BMI in the BELFAST study (r2 = 0.36; p = 0.03), though to date this has not been fully explored (Figure 1).

#### Genetics and NK cells

The functions of human natural killer (NK) cells are controlled by diverse families of antigen receptors. Prominent among these are the killer cell immunoglobulin-like receptors (KIR), a family of genes clustered in one of the most variable regions of the human genome. The KIR genes can be classified into A or B haplotypes, with A having a more inhibitory role, compared to the more activating role of B, on NK cell function. Considering that KIR genes might have an influence in longevity, we assessed KIR gene frequency in BELFAST nonagenarians but noted no differences in the major or grouped allele frequencies [19], though the study may have been underpowered, for the large number of KIR haplotypes identified.

In addition to genetic diversification, the KIR gene complex also shows differences at the functional level with different alleles having different protein expression levels and different avidity with their HLA ligand. We grouped BELFAST nonagenarians by A and B KIR haplotypes and showed in a preliminary study that there was a predominance of the pro-inflammatory cytokines with the B group of KIR genes [20]. We consider that this may be important in explaining, the pro-inflammatory background found with increasing age or inflamm-ageing [21], and could provide an explanation for, or be a consequence of, the increased number of NK and NKT-related cells, found in the very old [16,17].

### Cell-Mediated Immunity

#### Lymphocyte subsets

BELFAST nonagenarians [22,23] showed a range of changes in lymphocyte subsets which seem to be related to their advanced age and generally replicate other studies [14,15]. CD3 lymphocytes were apparently unchanged in number, though CD4 naïve cells decreased in our cross-sectional study, in both male and female nonagenarians, compared to younger cohorts [22]. We found that CD8 cell numbers remained unchanged for BELFAST 'elite' nonagenarians unlike other studies where CD8 cells appeared to accumulate with age, though subject groups may not be 'elite', as with us. Recent studies suggest that CD8 T cells may be age-sensitive by at least two partially independent mechanisms: fragile homeostatic control and gene expression instability in a large set of regulatory cell surface molecules [24].

#### CD4 lymphopenia and nutrition

Lymphopenia, < 1.5 × 10^9^, is considered a marker for protein energy malnutrition in children and adults [25]. In BELFAST nonagenarians, in keeping with others [26-28], we noted a fall in CD4 count with increasing age [22] and this has been reported as a fairly universal association with very advanced age. A decline in CD4 count, a rise in CD8 compartment and an increase in the NK cell numbers has been considered as a 'remodelling' of the immune compartment with ageing.

In BELFAST subjects, we had previously shown an association between the CD4 count and some nutritional markers [29], suggesting that potentially reversible nutritional and/or inflammation-related factors could be responsible for the CD4 lymphopenia in our aged group. From France and Japan other researchers also reported similar unexplained CD4 lymphopenia in 80 year olds [30,31].

**Figure 2:**
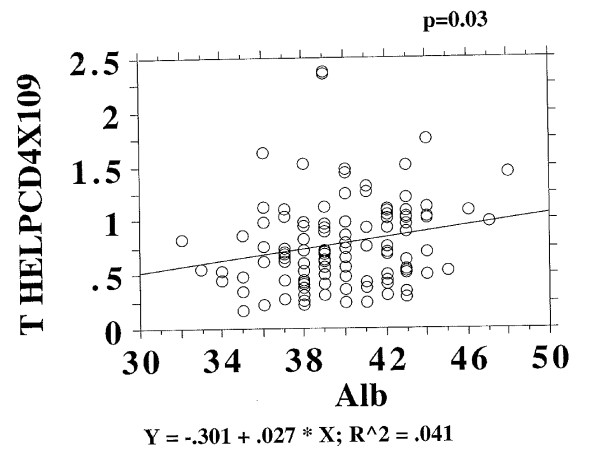
**CD4 count and Albumin in BELFAST study**.

In the BELFAST group the CD4 count was weakly and negatively correlated to albumin levels, though many older subjects in the BELFAST study also showed CD4 lymphopenia, where albumin values were well within the normal range Figure 2. These findings raised the possibility in our thinking, that nutrition and/or inflammation might play a part in CD4 lymphopenia associated with ageing.

#### CD4/CD8 Ratio and Ageing

**Figure 3:**
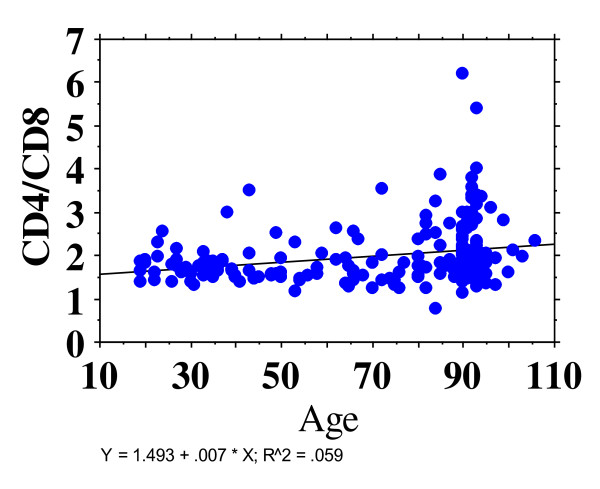
**CD4/CD8 ratio in BELFAST study**.

In 2008, Wikby's group in their NONa study [32] suggested that low CD4/CD8 ratio was a biomarker for reduced survival. Factors associated with Immune Risk Profile (IRP) were an associated cytomegalic virus index and a clonal predominance in subsets. In contrast to Wikby's finding, a preliminary analysis in BELFAST nonagenarians showed that the CD4/CD8 ratio was well maintained Figure 3 with increasing age [33]. However, to date, cytomegalic virus markers have not been measured in the BELFAST study and this would be important information to help interpret the different findings. Lately, the Wikby group have also reported findings of no change in CD4/CD8 ratio for centenarian survivors [34] arguing that centenarians are a special group, similar to findings in 'elite' BELFAST study subjects. The jury therefore remains 'out' as to whether a low or declining CD4/CD8 ratio in elderly people is a poor prognostic sign, or whether it is potentially reversible, if nutrition and/or infection resolve.

### Cytokines -Serum and Supernatant

Ageing is associated with an increased pro-inflammatory profile which was described by Franceschi as INFLAMM-AGEING [21]. The BELFAST study generally showed similar changes with an increase in pro-inflammatory serum cytokines i.e. IL-6, IL-12 and TNFα. In addition BELFAST subjects showed an accompanying increase in soluble IL-6 receptor and the IL-12p40 fraction. This suggested that the soluble cytokine factors both reduce and buffer the biological effect of their respective cytokines [35-37]. These effects were equally present in male and female elderly subjects. In general, the anti-inflammatory cytokines showed less change with increased age, in that IL-10 showed a modest increase, whereas the anti-inflammatory and 'active' form of TGFβ showed no apparent age-related change [35].

We wondered where the site of production of the increased cytokine profile might be located, since the finding had been replicated across several groups. In later work we showed that IL-6, IL-2 and IL-12 were produced spontaneously, not only by separated monocytes [35-37], but also from lymphocytes. We subsequently set up a methodology for intracellular identification of cytokines and demonstrated that IL-2, IL-6 and TNFα were produced from intracellular domains in stimulated lymphocytes [38].

#### Genes and Cytokines

Cytokine genes control cytokines and act like immune hormones to orchestrate the serum and tissue levels of the cytokines which drive the immune response. Cytokine polymorphisms have functional effects and determine serum cytokine responsiveness to stressors, including exercise and infections [39,35]. In early work on cytokine genes and the effect of genes on cytokines, we were the first to show that the IL-6 gene not only affects IL-6 production through its C and G alleles, but also affects the anti-inflammatory partner IL-10 and soluble IL-6 receptor in a reciprocal fashion [37]. This suggests that cytokine alleles which are high cytokine producers may also produce higher levels of their reciprocal soluble receptors and their partner anti-inflammatory cytokines and that the acute 'stress' responses are highly buffered and controlled in life, when people are well. It is possible that as people become older, the homeostatic 'immune thermostat' and buffering mechanism becomes blunted, responses become less focused with the timing out of sequence, and this leads to low grade chronic inflammation, and age-related disease.

In 2003 we were one of the first groups to conduct a large scale study into cytokine gene polymorphisms [39,40]. We argued that cytokine genes, in controlling and modulating immune responsiveness, might be important in longevity and show a change in polymorphism allele frequency in old age. Together with others we hypothesised that cytokine gene alleles, associated with a strongly pro-inflammatory response, could contribute to survival from childhood infections, but might drive immune activation and increase age-related disease [40]. Conversely cytokine polymorphisms which increased the anti-inflammatory profile, might well contribute to a more modulated response to mid life '*stressors' *and age-related disease and contribute to better quality ageing [41].

The BELFAST nonagenarian group, showed some attrition of the IL-6 GG allele in very aged persons, but otherwise gene allele frequencies for most of the cytokine polymorphisms were largely unchanged with longevity [39]. A major criticism could be that the study was insufficiently powered and in an attempt to address this on a larger scale, a meta-analysis of the effect of IL-6 and longevity was carried out, across some centres in Europe. This study suggested that IL-6 has different allele frequencies across Europe, with a north to south axis. There may also be differential effects of the G/C alleles across countries, since only the 3 Italian centres showed an association between the G allele and longevity [42].

More and larger cytokine gene studies need to be carried out since there is evidence that cytokine alleles produce different functional effects dependent upon tissue and cellular milieu [43,44]. The IL-6 gene may also have an environmental trigger or inhibitor which can modify or modulate its role. It has been postulated that IL-6 production and gene switching might be modulated by the Mediterranean diet as has been noted in cellular studies [45,46] with similar changes suggested as contributing to differences between Northern and Southern European incidence, and mortality from vascular disease. However early human studies have not provided much support for this hypothesis [47], though there is evidence that a 'Mediterrean' type diet may reduce the incidence of mortality from cardiovascular disease [48]. Larger studies and meta-analyses are therefore needed to circumvent the power issues, and should be addressed in larger genetic and life-style research such as the EU **Ge**netics of **H**ealthy **A**geing study [49].

### Cardiovascular Risk and BELFAST Nonagenarians

Several major factors associated with cardiovascular risk have been measured in the BELFAST study and some are discussed in relation to nutrition and genetic profile.

• **Blood Pressure **and anthropometric and dietary variables

• **Cholesterol**, High Density Lipoprotein (HDL), Low Density Lipoprotein (LDL)

• **Genotypes **previously related to increased cardiovascular risk

#### Blood Pressure

##### Blood Pressure and Anthropometric Variables

Blood pressure is the most important risk factor for heart disease and stroke [50]. In BELFAST nonagenarians, mean blood pressure was 137 and 84 mmHg for systolic and diastolic blood pressure respectively, with no difference between males and females (p = 0.57; p = 0.48 respectively). The mean BMI for both male and female nonagenarians was approximately 24.5 with no sex-related difference (p = 0.79).

In the BELFAST study, we found that tertiles of blood pressure were variously associated with BMI, weight and skin fold thickness in the nonagenarian cohort, so that higher weight, waist measurements and skinfold thickness increased the chance of having a blood pressure above 140/90 mmHg by about 30% [2]. This surprising finding is similar to findings in younger people, where obesity tracks with hypertension and often with the metabolic syndrome [51,52], and suggests that comparable pathogenic mechanisms may link blood pressure to weight-related variables even in 90 year olds.

Although obesity is considered a major risk factor for heart disease there seems to be paradox in that it also seems protective in a range of cardiovascular-related diseases [53]. A number of papers have noted that obese people with hypertension have lower mortality or stroke despite less effective blood pressure control. The mechanism for this finding is not known. In our hands, BELFAST nonagenarians seem to show a positive relationship between measures of weight and blood pressure, but with normal glucose [2], although the longer term outcome for these nonagenarians is not yet known.

Obesity may be different diseases in different people and the lean male with a family history of heart disease may have a higher cardiovascular risk, compared to the weightier female who has reached 89 years. Because of our findings, we do not suggest that 90 year olds should lose weight, but rather we raise the question as to whether weight and blood pressure have different trajectories in the 'oldest old', and whether genes related to obesity and/or blood pressure might be differentially expressed in the 'oldest old' in a sort of pleiotropic antagonism [54].

##### **Blood Pressure and Sodium**

**Figure 4:**
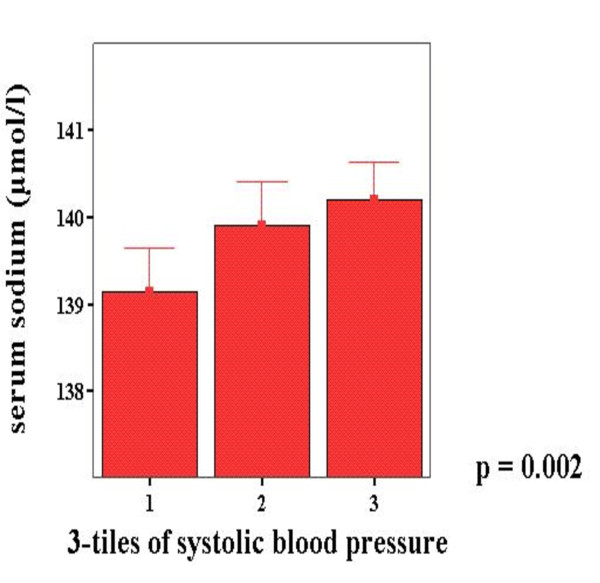
**Tertiles of Systolic and Diastolic Blood Pressure in relation to Serum Sodium in BELFAST study**. Systolic Blood Pressure. Tertile 1 < 120 mmHg. Tertile 2 120-140 mmHg. Tertile 3 > 140 mmHg.

The 24 hour dietary intakes were analysed by CompEat [CompEat nutritional analysis program, Lifeline Nutritional Services, London]. This showed a relatively low sodium intake at approximately 2 grams daily (1836 mg for females and 2087 mg for males), compared to population averages of 4-7 grams daily, and this finding may contribute to the relatively normal blood pressure found in the BELFAST nonagenarian group [2]. Increasing tertiles of serum sodium, although only weakly associated to sodium intake by dietary recall [r^2 ^= 0.27; p = 0.046], were significantly correlated with increasing tertiles of blood pressure in BELFAST nonagenarians (Figure 4).

This is of special interest because Feng & McGregor have demonstrated that lower salt intake is helpful in lowering blood pressure measurements population-wide [55]. Previous studies also suggest that blood pressure can be optimised in people over 65 years with reduced salt intake, since there is considered to be increased sensitivity to salt restriction [56]. It is not known whether this is a direct effect of sodium intake on vascular resistance or a more complex gene/environment-related effect [57], which like weight, may be differently expressed in the 'oldest old'

#### Cholesterol, HDL and LDL fractions

A couple of interesting points emerged from 24 hour dietary intake by CompEat [CompEat nutritional analysis program, Lifeline Nutritional Services, London]. Overall the total calorie intake was relatively low at approximately 1426 Kcals, with males taking about 200Kcals more than female nonagenarians. According to CompEat, the average fat total for nonagenarians was approximately 57 g distributed as 37 g of saturated fat content, 4 g polyunsaturates, and 16 g monosaturates. Although the total dietary fat total could be considered low normal, the distribution shows a higher saturated fat percentage, than might be considered optimal.

Individual serum cholesterol levels and HDL and LDL fractions are both genetically and nutritionally determined. BELFAST nonagenarians showed a mean cholesterol value around 5.3 umol/l for both male and female nonagenarians, which would be considered low normal for the local Northern Ireland population, in the absence of treatment. HDL however, was quite well preserved in both male and female nonagenarians with median values of 1.3 umol/l. for female and over 1.umol/l for male nonagenarians, though some males had values < 1.0 umole/l which would be considered less than optimal. The HDL/cholesterol ratio at around 3.5 is reasonably satisfactory in a group with no major risk factors, other than advanced age [58].

However cholesterol and its fractions are dictated by several genes, the most well recognised of which, is ApoE, with the ApoE 4 allele being associated with higher cholesterol and atherosclerotic clinical risk [59-61]. BELFAST nonagenarians who carried the ApoE4 allele showed higher serum cholesterol values compared to those who carried the ApoE2 allele. The frequency of ApoE4 allele carriage was reduced by 50% in nonagenarians, compared to the local < 65 year old population [62], suggesting that vascular-related risk and early mortality may have had an attrition effect on the ApoE4 gene pool in 90 year old survivors.

#### Genes-Apolipoprotein E gene (ApoE), Angiotensinogen Converting Enzyme gene (ACE) and MethylenetetrahydrofolateReductase Gene (MTHFR)

These three genes have alleles ie ApoE4 for ApoE, DD for ACE and tt for MTHFR, which have been associated with premature vascular-related disease, [63-65], including dementia [66,67]. It has been argued that carriers of these genes have increased risk of early vascular-related death, whether from heart disease or dementia, and this would be represented in nonagenarian survivors with attrition of the 'risk' allele frequency, compared to younger age groups.

In keeping with this hypothesis, BELFAST nonagenarians, showed a fall in ApoE4 allele frequency reduced from 16% in < 65 year olds from the same geographical area, enlisted for the **MONI**toring of **CA**rdiovascular (MONICA) project, down to 8% in BELFAST nonagenarians. There was a reciprocal change in ApoE2 rising to 12% in nonagenarians compared to 8% in < 65 year old MONICA local subjects [62]. A similar decrease in ApoE4 allele frequency has been consistently noted in nonagenarian and centenarian survivors across many European and world-wide populations, suggesting that the BELFAST study is '*fit for purpose*' and has replicated other findings [68-70].

The DD allele previously associated with early myocardial infarction and death [64] was not significantly changed in frequency in BELFAST nonagenarians and therefore does not add support to the suggestion that homozygous DD carriage tracks with early mortality, at least for BELFAST nonagenarians [71]. Although the D allele of ACE might be expected to increase blood pressure, this was not the case for BELFAST octo/nonagenarians and in other studies

BELFAST octo/nonagenarians also showed a non-significant reduction in frequency of the tt allele of MTHFR [72], which is associated with higher homocysteine, and considered a vascular risk factor, with increased mortality [65,73]. However the interesting and important effect that dietary folate has in lowering homocysteine in t allele carriers, was demonstrated to be present in octo/nonagenarians in the BELFAST study [1]. Here a powerful gene-nutrition effect is exemplified where dietary folate consumption from green vegetables at a personal level, or in fortification of flour at a population level, can reduce homocysteine in t allele carriers, with the potential to reduce vascular risk [74].

In a multiple regression analysis to assess the relative importance of these 3 genes with respect to longevity in BELFAST octo/nonagenarians, ApoE was the most significant gene, with the ApoE 2 allele contributing positively, though in a modest way, to nonagenarian longevity. The ApoE 4 allele conversely, demonstrated a negative effect with respect to survival beyond 90 years [74]. Previously, Heijmans [75] had suggested that ApoE did not contribute to cardiovascular mortality in old age in the Leiden study, but did contribute significantly to Alzheimers dementia. Locally we were unable to replicate this finding, and found no difference in nonagenarian mortality outcomes to time of death for ApoE 2 compared to ApoE4 BELFAST nonagenarians or in relation to cause of death, as documented by death certificate [74].

## Summary

Nonagenarians and centenarians are of intense interest to scientists, since they may help us understand how to age not only longer, but better. Ageing better is important to each of us personally but is of enormous interest to our governments, who recognise that healthy ageing has a huge economic dividend for society.

BELFAST 'elite' nonagenarians show evidence of a competent immune system, programmed with increased NK cells to scan presumably for '*damaged' *cells and all sorts of intruders. There seems to be increasing evidence that diet and environment can influence the immune system and the cytokine milieu, and can modulate the body's defence to every type of stressors-from infections to cancer.

Cardiovascular risk is also influenced by what we eat, or even by what our grandmother and mother ate [76,77]. The old saying that '*what you eat is what you are' *seems increasingly to be true, as we begin to dissect out how our genes can be modified before birth, and by our mother's nutritional opportunities. Evidence from the Dutch famine cohort provides a clear association between the later cardiovascular health of offspring and the mother's nutritional status during pregnancy [77].

In ageing and age-related disease, there is increasing evidence that our genes which we inherit are our 'genetic capital', and we increasingly understand that gene expression and their effects are influenced by our environmental choices, whether they be nutritional, exercise or stress-related, or probably also our sex-hormones. Diet and lifestyle choices are therefore key to helping our genes facilitate better longer ageing, if we are lucky enough to start off with good genetic 'capital'. Besides our genes, good nutritional support and life style choices may be able to maximise our positive genetic attributes and minimise any damaging effects.

Like any wise strategist or fiscal planner, we each need to take control of our destiny and plan to age better.

## Competing interests

The author has no conflict of interest nor any financial or other interest associated with this research work.
